# A joint model of household time use and task assignment for elderly couples with multiple constraints

**DOI:** 10.1371/journal.pone.0247187

**Published:** 2021-03-11

**Authors:** Kangning Zheng, Enjian Yao, Yongsheng Zhang

**Affiliations:** 1 School of Traffic and Transportation, Beijing Jiaotong University, Beijing, China; 2 Key Laboratory of Transport Industry of Big Data Application Technologies for Comprehensive Transport, Beijing Jiaotong University, Beijing, China; The University of Tokyo, JAPAN

## Abstract

Household time-use patterns are expected to reflect each household member’s daily activity participation and duration with intra-household interactions constrained by multiple budgets. Among various activities, the allocated activity derived from undertaking a household task is studied relatively less in the literature. Who will take an allocated activity is a discrete choice problem of household task assignment, and once a household member is assigned with one household task, other members will have more time to do other activities. To better understand household time-use patterns affected by household task assignment, this paper proposed a joint household-level multiple discrete-continuous extreme value-multinomial logit (MDCEV-MNL) model with multiple constraints. The Karush-Kuhn-Tucker (KKT) method combined with the simulation-based maximum likelihood estimation method is proposed to estimate the proposed model. Based on the household activity-travel data from Beijing of China, the proposed model is customized to explore elderly couples’ time-use patterns with intra-household interactions affected by household task assignment. Following the findings, policy implications are suggested to build an age-friendly society.

## 1. Introduction

Population aging issues are significantly affecting the sustainability of economic development and society’s stability [1]. Nowadays, the increasing elderly population has attracted the attention of social sciences concerning social exclusion [2]. In order to avoid social exclusion, many efforts have been done to encourage elderly people to undertake more out-of-home activity participations and durations. Due to retirements and aging, activity-travel behaviours between young people and elderly people are found to be different [3–5]. However, existing research about elderly people’s time-use patterns ignored impacts of household task assignment in a household.

In the household level, the intra-household interactions of the elderly couple complex the elder’s daily time use, since the couple’s daily life will always be filled with the cooperation, negotiation and compromise [6]. Elderly couples’ task assignment and time use are the trade-off outcomes among household members, considering household requirements and available resources, individual preference and emotional connection. Household task assignment is a decision process of assigning one household member with one household task, which is regarded as a discrete choice problem. Household time-use patterns refer to the participations and durations of daily activities for household members. It is a multiple discrete-continuous choice problem, since each member may undertake more than one daily activity given a time budget. From a household perspective, associated with participants, out-of-home activities can be classified into three groups: independent, joint and allocated activities [3]. An independent activity refers to the activity (which is not a household task) undertaken by oneself, such as work, school, personal business, etc. A special property of an independent activity is that the participant cannot be substituted by others, which means the activity must be undertaken by him/herself. The joint activity refers to the activity undertaken by the elderly couple together. For example, if an elderly couple goes somewhere for leisure together or go shopping jointly, we call it joint leisure or joint shopping. An allocated activity is associated with a household task (e.g. shopping for household maintenance, escorting school children), which means the activity could be undertaken by any household member assigned with the household task. A major difference between independent and allocated activities is that the participant of an allocated activity can be substituted by other household members. If one household member undertakes an allocated activity (i.e., a household task), other members save time to do other activities (i.e., they don’t need to do the same household task). In the literature, allocated activities are less explored compared with independent and joint activities.

Furthermore, behaviors and decision-making processes behind household time use and task assignment for elderly couples in China are different, compared with other countries, due to the incomplete social welfare and public service as well as the traditional Chinese family values [7]. The values are the response of Confucianism, whose core idea behind family cohesion is the encouragement of cooperation and coordination. As a result, many elderly couples would like to help their adult-children to undertake household tasks due to their more flexible timetable [4,8]. Especially for the school children who cannot travel between school and home independently, escorting is required due to the low penetration of school buses and some safety issues in China [9].

This paper will shed light on developing a joint model of household time use and task assignment with intra-household interactions and multiple constraints to explore impacts of household task assignment on elderly couples’ time-use patterns in Beijing, China.

The remaining sections are organized as follows. Section 2 is ‘Literature Review’. Then, a general household time-use model integrating task assignment with multiple constraints is formulated in Section 3. The estimation approach to the proposed integrated model is provided in Section 4. Section 5 empirically evaluates the proposed model and provides discussions about policy implications. The last section is ‘Conclusion’.

## 2. Literature review

Since travel behaviors are derived from out-of-home activities, activity-based approaches have attracted transportation researchers’ interests [10–12]. Time-use modelling [13], which focuses on the joint of multiple discrete activity participations and continuous durations, is one of the activity-based approach tasks. In the past decades, plenty of individual-based time-use models have been pushed out based on various modelling frameworks for multiple discrete-continuous choices [14–20]. For more information, please refer to a review of time-use modelling [21]. Existing research of multiple discrete-continuous choices for individual time-use modeling can be classified into two groups: multivariate discrete-continuous frameworks (e.g., [19,22–26] and Karush-Kuhn-Tucker (KKT) demand systems (e.g., [22,27–31]).

Among various modelling frameworks, the KKT-based multiple discrete-continuous extreme value (MDCEV) model structure [22,28] is more attractive. The model can capture the principle of diminishing marginal utility with increasing consumption and offer a closed-form probability expression for easier estimation and application. Based on the MDCEV structure, various extended models have been widely seen in the literature. There are three major types of extensions: 1) Towards flexible stochastic utility. For example, by relaxing the assumption of independent error distribution among alternatives, MDCNEV [32], MDCGEV [33], MDCP [34] models were proposed; by allowing for unobserved heterogeneity, LC-MDCEV [35], LC-MDCP [36] models were proposed. 2)Towards flexible patterns [30,37]. There may be three major patterns among alternatives: complementarity, imperfect substitution and perfect substitution. 3)Towards flexible constraints, for example, multiple constraints [31] and stochastic time budget [25].

However, all above-mentioned models focus on individual-based time-use patterns. The intra-household interactions are ignored. Apparently, household members have intra-household interactions in terms of joint activity participations, household resources allocations and tasks assignments [6,38–41]. Therefore, modelling time use has been shifting from individual-level to household-level (e.g., [3,5,8,42,43]).

According to involved participants and their different intra-household interactions, household activities can be classified into joint, independent and allocated activities [3,6,18]. The study on the joint activity, which typically reflects the intra-household interactions among household members, dominates household activities-related research [8,44–47]. The allocated activity is usually not addressed or equivalent to the independent activity in existing research [5,6], since only one household member participates in the activity. Who will be assigned to undertake an allocated activity is substitutable, which means any household member can undertake the allocated activity, whereas, the independent activity, such as work and personal business, can only be undertaken by oneself.

Actually, the allocated activity is the outcome of integration between household task assignment and time use. For example, a schoolchild may require to be escorted by one adult household member on school trips [48]. Safety concerns about school children have significant effects on household members’ activity participation and time use, especially for the one who is assigned to escort [49]. The escort activity can be regarded as a household task. Then by negotiation, one of the household members will be assigned to undertake this escort activity. In the literature, researches on household task assignment have been done by decades (e.g., [19,50]), including but not limited towards childcare [51], escorting school child for school [52], maintenance shopping [17], etc.

Generally speaking, household task assignment will affect household time use. In a household, the one assigned to undertake a household task will have less time for other activities, whereas the others within a family may have more available time as they escape from the household tasks. On the contrary, each household member’s available time for household tasks will be compared when making the decisions of household task assignments. Simply, the one with more flexible time may be better to take the responsibilities for household tasks. Impacts of household task assignment on household time use should be paid more attention to.

Due to the intensity of population aging problem, many researchers have focused on the elder’s activity and travel patterns [2,53,54], since elderly couples usually have different preferences towards activities and travels compared with young couples [5]. The Chinese elderly couples would like to help their adult-children undertake some household tasks [4,55], such as escorting children for school, shopping for household maintenance, etc. Especially for the activity of escorting school children, it is a typical allocated activity with Chinese characteristics. As the elderly couple usually has more free time than the young couple, one of the elderly couple will be assigned to escort the child for school so that the young couple could concentrate on work [4,8]. Since an allocated activity is derived from household task assignment, in order to have a better understanding of elderly couples’ time-use patterns, impacts of household task assignment should be incorporated into a household-level time-use model. Household task assignment is usually regarded as a discrete choice problem [17,48,52]. However, existing research about the relationships between time use and task assignment usually assumed that the participant for an allocated activity had been assigned in advance so that the decision process of task assignment was ignored [3,6].

Therefore, this paper tried to incorporate the decision process of task assignment into household time-use model to involve the interactions between time-use and task assignment. As mentioned earlier, a household-level MDCEV model is preferred to explore household time-use patterns. Meanwhile, a MNL model is utilized in this paper to formulate the discrete choice process of household task assignment. By integration, a joint household-level MDCEV-MNL model is proposed by this paper to explore the impacts of household task assignment on household time-use patterns.

The joint MDCEV-MNL framework has already been found in the literature. For example, Bhat (2009) [56] and Sudeshna (2006) [57] applied the framework to analyze simultaneous choice of vehicle holdings and usage. Pinjari (2009) [58] applied the framework in the choice of residential location and time use. However, existing research about MDCEV-MNL model only concerns a single constraint. For household time use and task assignment, each household member will have its time budget. It means the objective is constrained by multiple constraints. Therefore, this paper proposes a household-level MDCEV-MNL model with multiple constraints.

The contributions of this research are concluded as follows:

1. This paper extends a joint MDCEV-MNL model with one constraint into with multiple constraints.
2. An estimation algorithm associated with the joint MDCEV-MNL model with multiple constraints is proposed.
3. This paper customizes the joint MDCEV-MNL model into the household time use and task assignment scenario. And household time use and task assignment patterns of elderly couples in Beijing, China are specified.

## 3. Methodology

Household time use is an optimization problem that pursues the maximal household utility of time-use for all household members’ all activities. It should be noted that the proposed household time-use utility function is constrained by multiple constraints, i.e., each household member’s time budget. As mentioned above, household out-of-home activities are classified as independent, joint and allocated activities in this paper. Considering impacts of household task assignment on household time-use patterns, an endogenous integration of household time use and task assignment will be implemented by this paper.

This section will first introduce how to build a general household utility function of time-use and then formulate the utility of different type of activities. Household task assignments associated with allocated activities will be endogenously incorporated into the household time-use model. Finally, an optimization model of household time-use integrating household task assignment with multiple constraints will be developed.

### 3.1 Household utility of time use

Considering the three types of household activities and all members’ available times, the general Household Utility Function (HUF) *U*_*g*_ can be formulated as a linear function for simplicity as follows.

$$U_{g}=U_{S}^{g}+U_{J}^{g}+U_{A}^{g}+U_{0}^{g}$$ (1)

where, for the household g, *U*_*g*_ is the total utility; $U_{S}^{g}$ is the utility of independent activity *s*; $U_{J}^{g}$ is the utility of joint activity *j*; $U_{A}^{g}$ is the utility of allocated activity *a*; *g*,*m*,*k* separately belongs to the sets of household (*g* = 1,2,…,G), household member (*m* = 1,2,…,*M*) and alternative activities (*k* = 1,2,…,*K*); $U_{0}^{g}$ is the utility of “outside goods”, that is the time unconsumed by the three types of activities (e.g., in-home time). The “outside goods” will be consumed by each of the household members definitely. The total utility of “outside goods” for the household *g* is formulated as a sum of the utility of the “outside goods” consumed by each member as follows.

$$U_{0}^{g}=\sum_{m}^{M}u_{m0}^{g}(t_{m0}^{g})$$ (2)

where $u_{m0}^{g}(t_{m0}^{g})$ is the utility of the “outside goods” consumed by member *m* for $t_{m0}^{g}$ time. The utility can be formulated as $u_{m0}^{g}(t_{m0}^{g})=\varphi_{m0}^{g}\ln(t_{m0}^{g}+1)$ according to MDCEV model [22,28], where only the error term appears in the baseline utility function $\varphi_{m0}^{g}=\exp(\varepsilon_{m0}^{g})$.

### 3.2 Utility for independent activity

Household members usually engage in one or more independent daily activities, e.g. personal business. Independent activity utility for household *g* is formulated by the sum of the utility of each activity attended by each household member as follows.

$$U_{S}^{g}=\sum_{m}^{M}\sum_{s}^{N^{S}}u_{sm}^{gS}(t_{sm}^{gS})$$ (3)

where $U_{S}^{g}$ is the utility of independent activity *s* which the member *m* in household *g* would like to participate in; $t_{sm}^{gS}$ is the duration for activity *s* and $t_{sm}^{gS}\geq 0$. $t_{sm}^{gS}=0$ means the member doesn’t do this independent activity. At this moment, the corresponding utility will be zero as well. Usually, activity duration is positively correlated with activity utility, which means activity utility will increase with the duration increasing. However, the increasing rate may decline due to the satiation effect. According to the MDCEV model [22,28], the utility of independent activity *s* in which the member *m* consumes $t_{sm}^{gS}$ time can be simplified as follows:

$$u_{sm}^{gS}(t_{sm}^{gS})=\varphi_{sm}^{gS}\ln(t_{sm}^{gS}+1)$$ (4)

where $\varphi_{sm}^{gS}$ is the baseline utility, representing the benefit of one unit of consumed time for activity *s*.

$$\varphi_{sm}^{gS}=\exp(\sum_{i}\beta_{smi}^{gS}X_{smi}^{gS}+\varepsilon_{sm}^{gS})$$ (5)

where *X* is the *i*-th individual or household attribute; $\beta_{smi}^{gS}$ is the corresponding parameter; $\varepsilon_{sm}^{gS}$ is the unobserved error term.

### 3.3 Utility for joint activity

Household members may participate in some activities together, namely, joint activities. The total utility of all joint activities for a household is a sum of each joint activity’s utility, as shown below.

$$U_{J}^{g}=\sum_{j}^{N^{J}}u_{j}^{gJ}(t_{j}^{gJ})$$ (6)

where $U_{J}^{g}$ is the utility of joint activity *j* for household *g*; $t_{j}^{gJ}$ is the joint duration.

Joint activity is associated with one of the typical intra-household interactions. By negotiation with each other, more than one household member will join in the same activity. The utility of joint activity can utilize the same structure to the utility function in the MCDEV model [22,28], as shown in the following Eq (7). But in order to represent intra-household interactions, the utility function should be revised into a joint utility function [5], where the baseline utility function is revised as a *Nash-type* multi-linear utility function [3], as shown in Eq (8).

$$u_{j}^{gJ}(t_{j}^{gJ})=\varphi_{j}^{gJ}\ln(t_{j}^{gJ}+1)$$ (7)

$$\begin{array}{l} \varphi_{j}^{gJ}=\prod_{m}\varphi_{jm}^{gJ}\\ \;=\exp(\sum_{m}\sum_{i}\beta_{jmi}^{gJ}X_{jmi}^{gJ}+\sum_{m}\varepsilon_{jm}^{gJ})\\ \;=\exp(\sum_{i=1}^{i^{\prime }}\beta_{ji}^{gJ}X_{ji}^{gJ}+\sum_{m}\sum_{i=i^{\prime }+1}^{I}\beta_{jmi}^{gJ}X_{jmi}^{gJ}+\varepsilon_{j}^{gJ}) \end{array}$$ (8)

where $\varphi_{j}^{gJ}$ is the baseline utility function for the joint activity *j*; $\varphi_{jm}^{gJ}$ is the baseline utility function for member *m* who is involved in the joint activity *j*; if *i*≤*i*′, $X_{ji}^{gJ}$ is the *i-th* household attribute, otherwise, $X_{jmi}^{gJ}$ is the *i-th* individual attribute; $\beta_{ji}^{gJ}$ and $\beta_{jmi}^{gJ}$ are the corresponding parameters; $\varepsilon_{jm}^{gJ}$ is the error part for member *m*; $\varepsilon_{j}^{gJ}$ is the sum of each member’s error, representing the error part for the joint activity.

### 3.4 Utility for allocated activity

In a household, someone may be a representative of the household to participate in an activity, such as maintenance shopping, escorting school children, etc. The allocated activity involves a decision-making problem of identifying which household member should be assigned to undertake the allocated activity. The substitutability among household members is the typical property of the allocated activity. For each allocated activity, we want to know whether it is undertaken or not and who will be assigned to undertake the activity and the corresponding duration. The latter one corresponds to the household task assignment. Note that this paper only considers the scene that only one member is assigned to complete the ‘task’ (e.g. one of the elderly couple escorting a school child) when talking about household task assignment. The allocated activity also reflects one type of intra-household interactions, which is *Maximum-type* [41].

The total utility of all allocated activities for a household is the sum of the utility of each allocated activity in this paper, as shown in Eq (9).

$$U_{a}^{g}=\sum_{a}^{N^{A}}u_{a}^{gA}(t_{a}^{gA})$$ (9)

where $U_{a}^{g}$ is the utility of allocated activity *a* for household *g*; $t_{a}^{gA}$ is the duration for allocated activity *a*. Then the duration structure of all allocated activities for household time uses can be described as $\left(t_{1}^{gA},\cdots ,t_{a}^{gA},\cdots ,t_{A}^{gA}\right)$.

For $u_{a}^{gA}$: the utility of each allocated activity, which is equivalent to the utility of the activity undertaken by the assigned household member. In order to incorporate household task assignments into household time uses and for an easier interpretation of the estimation process in the following section, we use the following summation equation to formulate the utility of each allocated activity by assuming a specific structure of the durations and a specific property of the utility function. The specialties will be introduced after the equation.

$$u_{a}^{gA}(t_{a}^{gA})=\sum_{m}^{M}u_{am}^{gA}(t_{am}^{gA})$$ (10)

where $u_{am}^{gA}(t_{am}^{gA})$ is the utility of allocated activity *a* undertaken by household member *m* respectively.

Note that the duration $t_{am}^{gA}$ has special characteristics. If an allocated activity should be done by one of the household members, the time $t_{am_{a}}^{gA}$ allocated to the activity *a* by an assigned member *m*_*a*_ can be expressed as $\left(0,\cdots ,t_{am_{a}}^{gA},\cdots ,0\right)$ from the household perspective, where *m*_*a*_ represents the assigned member to attend allocated activity *a*. It means the duration of an allocated activity is only occupied by the assigned member, while duration is zero for other members, namely, $\left(t_{a1}^{gA},\cdots ,t_{am}^{gA},\cdots ,t_{aM}^{gA})=(0,\cdots ,t_{am_{a}}^{gA},\cdots ,0\right)$ for each allocated activity. And if the duration $t_{am}^{gA}$ is zero, the time $t_{am_{a}}^{gA}$ will be zero and the utility $u_{am}^{gA}(t_{am}^{gA})$ will also be zero. Then for all allocated activities, household time use can be augmented as a nested structure $\left(\left(0,\cdots ,t_{1m_{1}}^{gA},\cdots ,0),\cdots ,(0,\cdots ,t_{am_{a}}^{gA},\cdots ,0),\cdots ,(0,\cdots ,t_{Am_{A}}^{gA},\cdots ,0\right)\right)$ from the original form $\left(t_{1}^{gA},\cdots ,t_{a}^{gA},\cdots ,t_{A}^{gA}\right)$, where the value of $t_{a}^{gA}$ is equal to the value of $t_{am_{a}}^{gA}$.

Via above augmentation [33], the household task assignments are incorporated into household time uses. The final results will simultaneously display the duration of an allocated activity *a* and the household member *m*_*a*_ who is assigned to participate in the activity. View this nested structure, we can clearly see the perfect substitutes in household task assignment process, since in each nest of the augmented structure (referring to one allocated activity), only one substitute’s value (i.e., the assigned household member’s time) may be greater than zero, whereas others are all zero. The imperfect substitutes in the household time-use process can also be seen, since more than one value may be greater than zero among all nests. We provide more detailed information about the integration of household task assignment and time use in the next section and the endogenous interactions between them.

The duration of one allocated activity can be zero, which means no household member implements the corresponding allocated activity. This paper develops an utility function of an allocated activity which is similar to the structure of the utility function in the MDCEV model [22,28], as shown below.

$$u_{am}^{gA}(t_{am}^{gA})=\varphi_{am}^{gA}\ln(t_{am}^{gA}+1)$$ (11)

$$\varphi_{am}^{gA}=\exp(\sum_{i}\beta_{ai}^{gA}X_{ai}^{gA}+\sum_{i}\beta_{ami}^{gA}X_{ami}^{gA}+\varepsilon_{am}^{gA})$$ (12)

where $\varphi_{am}^{gA}$ is the baseline utility for allocated activity *a* undertaken by member *m*; $X_{ai}^{gA}$ is the household-level attribute for household time use; $X_{ami}^{gA}$ is the individual-level attribute for household task assignment; $\beta_{ai}^{gA}$ and $\beta_{ami}^{gA}$ are the parameters; $\varepsilon_{am}^{gA}$ is the unobserved error term. In additionally, the error terms of allocated activities in household-level also have a nested structure, that is

$$\varepsilon^{gA}=\left(\left(\varepsilon_{1,1}^{gA},\cdots ,\varepsilon_{1m}^{gA},\cdots \varepsilon_{1M}^{gA}),\cdots ,(\varepsilon_{a1}^{gA},\cdots ,\varepsilon_{am}^{gA},\cdots \varepsilon_{aM}^{gA}),\cdots ,(\varepsilon_{A1}^{gA},\cdots ,\varepsilon_{Am}^{gA},\cdots \varepsilon_{AM}^{gA}\right)\right).$$

The nested structure of the error terms is the integration of household time use and task assignment. The perfect substitution will be performed in each nest, whereas the imperfect substitution will be performed across the nests.

After incorporating household task assignment into household time use, the household-level utility function of an allocated activity can be described as follows.

$$u_{a}^{gA}(t_{a}^{gA})=\varphi_{a}^{gA}\ln(t_{a}^{gA}+1)$$ (13)

$$\varphi_{a}^{gA}=\exp(\sum_{i}\beta_{ai}^{gA}X_{ai}^{gA}+\psi_{a}^{gA})$$ (14)

where $\psi_{a}^{gA}$ is the random composite utility of household task assignment. If the random composite utility function is derived from maximization the utility of household task assignment, the *Maximum-type* [41] can be obtained. The derivation process can be found in the “Model estimation” section.

### 3.5 Optimization of household time use

Since each involved member’s time budget is limited, we have the following objective household utility function (HUF) with multiple constraints.

$$Max\;U_{g}=\sum_{m}^{M}u_{m0}^{g}(t_{m0}^{g})+\sum_{m}^{M}\sum_{s}^{S}u_{sm}^{gS}(t_{sm}^{gS})+\sum_{j}^{J}u_{j}^{gJ}(t_{j}^{gJ})+\sum_{a}^{A}\sum_{m}^{M}u_{am}^{gA}(t_{am}^{gA})$$ (15)

$$s.t.\;\sum_{s}^{S}t_{sm}^{gS}+\sum_{j}^{J}t_{j}^{gJ}+\sum_{a}^{A}t_{am}^{gA}+t_{m0}^{g}=T_{m}^{g},\;m=1,\cdots ,M$$ (16)

where, *HUF* is increasing and continuously; $u_{m0}^{g}(t_{m0}^{g})$ is household *g*’s utility of “outside goods” as mentioned in Eq (1); $u_{sm}^{gS}(t_{sm}^{gS})$ is household member *m*’s utility of partaking in an independent activity *s*; $u_{j}^{gJ}(t_{j}^{gJ})$ is the utility of joint activity *j*, where the elderly couple is involved simultaneously; $u_{am}^{gA}(t_{am}^{gA})$ is the assigned household member *m*’s utility of partaking in the allocated activity *a*; *t* denotes time-use in different types of activities; $T_{m}^{g}$ is the individual *m*’s time budget (e.g. 24 hours). The above optimization problem will be solved in the next section.

## 4. Model estimation

The Karush–Kuhn–Tucker (KKT) method is commonly used to estimate individual-level and household-level time uses [5,50]. By Lagrangian transformation, the following Lagrangian function is obtained.

$$L=U_{g}-\sum_{m=1}^{M}\left[\lambda_{m}\left(\sum_{s}^{S}t_{sm}^{gS}+\sum_{j}^{J}t_{j}^{gJ}+\sum_{a}^{A}t_{am}^{gA}+t_{m0}^{g}-T_{m}^{g}\right)\right]$$ (17)

The KKT first order conditions of the Lagrangian function for the optimal time uses under multiple constraints are derived according to existing research [5,22].

The “outside goods” will be consumed definitely, that is $t_{m0}^{g*}>0$ for all members in all households. Each Lagrangian multiplier *λ*_*m*_ can be derived based on the consumed “outside goods” $t_{m0}^{g*}$ of each household member.

$$\lambda_{m}=\frac{\exp(\varepsilon_{m0}^{g})}{t_{m0}^{g*}+1}$$ (18)

For the independent activities, the KKT first order conditions are shown as below after logarithmic transformation.

For the independent activities,

$$\varepsilon_{sm}^{gS}=V_{sm}^{gS},\;if\;t_{sm}^{gS*}>0$$ (19)

$$\varepsilon_{sm}^{gS}=V_{sm}^{gS},\;if\;t_{sm}^{gS*}>0$$ (20)

where $V_{sm}^{gS}=\varepsilon_{m0}^{g}-\ln\left(t_{m0}^{g*}+1\right)-\sum_{i}\beta_{smi}^{gS}X_{smi}^{gS}+\ln(t_{sm}^{gS*}+1)$

For the joint activities,

$$\varepsilon_{j}^{gJ}=V_{j}^{gJ},\;if\;t_{j}^{gJ*}>0$$ (21)

$$\varepsilon_{j}^{gJ}<V_{j}^{gJ},\;if\;t_{j}^{gJ*}=0$$ (22)

where $V_{j}^{gJ}=\ln\left(\sum_{m}\frac{\varepsilon_{m0}^{g}}{t_{m0}^{g*}+1}\right)-\sum_{m}\sum_{i}\beta_{jmi}^{gJ}X_{jmi}^{gJ}+\ln(t_{j}^{gJ*}+1)$

For allocated activities, some specialties should be introduced. As mentioned in **3.4**, the duration of an allocated activity is augmented to a nest of the duration of each member’s participation, that is $t_{a}^{gA}\to (0,\cdots ,t_{am_{a}}^{gA},\cdots ,0)$. If an allocated activity *a* is undertaken, the duration will be equal to the one consumed by the assigned member *m*_*a*_ while the durations of other members will be zero. The property of perfect substitutions makes only one at most be greater than zero in the nest of durations. In other words, no matter which member’s consumed time for an allocated activity is greater than zero, the consumed time is the duration of the activity. Thus, we can get a set of KKT first order conditions.

$$\left[\begin{array}{l} \sum_{i}\beta_{ai}^{gA}X_{ai}^{gA}-\ln(t_{a}^{gA*}+1)+\sum_{i}\beta_{a1i}^{gA}X_{a1i}^{gA}+\varepsilon_{a1}^{gA}+\ln\left(t_{1,0}^{g*}+1\right)-\varepsilon_{1,0}^{g}=0,\;if\;t_{a1}^{gA*}>0\\ \vdots \\ \sum_{i}\beta_{ai}^{gA}X_{ai}^{gA}-\ln(t_{a}^{gA*}+1)+\sum_{i}\beta_{ami}^{gA}X_{ami}^{gA}+\varepsilon_{am}^{gA}+\ln\left(t_{m0}^{g*}+1\right)-\varepsilon_{m0}^{g}=0,\;if\;t_{am}^{gA*}>0\\ \vdots \\ \sum_{i}\beta_{ai}^{gA}X_{ai}^{gA}-\ln(t_{a}^{gA*}+1)+\sum_{i}\beta_{aMi}^{gA}X_{aMi}^{gA}+\varepsilon_{aM}^{gA}+\ln\left(t_{M0}^{g*}+1\right)-\varepsilon_{M0}^{g}=0,\;if\;t_{aM}^{gA*}>0 \end{array}\right]$$ (23)

$$\left[\begin{array}{l} \sum_{i}\beta_{ai}^{gA}X_{ai}^{gA}-\ln(t_{a}^{gA*}+1)+\sum_{i}\beta_{a1i}^{gA}X_{a1i}^{gA}+\varepsilon_{a1}^{gA}+\ln\left(t_{1,0}^{g*}+1\right)-\varepsilon_{1,0}^{g}<0,\;if\;t_{a1}^{gA*}=0\\ \vdots \\ \sum_{i}\beta_{ai}^{gA}X_{ai}^{gA}-\ln(t_{a}^{gA*}+1)+\sum_{i}\beta_{ami}^{gA}X_{ami}^{gA}+\varepsilon_{am}^{gA}+\ln\left(t_{m0}^{g*}+1\right)-\varepsilon_{m0}^{g}<0,\;if\;t_{am}^{gA*}=0\\ \vdots \\ \sum_{i}\beta_{ai}^{gA}X_{ai}^{gA}-\ln(t_{a}^{gA*}+1)+\sum_{i}\beta_{aMi}^{gA}X_{aMi}^{gA}+\varepsilon_{aM}^{gA}+\ln\left(t_{M0}^{g*}+1\right)-\varepsilon_{M0}^{g}<0,\;if\;t_{aM}^{gA*}=0 \end{array}\right]$$ (24)

Above equalities and inequalities could be put into a set of KKT first-order conditions due to the integration of household task assignment. Since the one with the maximum utility will be assigned to participate in the allocated activity, the above conditions can be condensed to a new set of equalities and inequalities by using the *max* function. After that, the equation is similar to the *Maximum-type* function [41].

$$\max\left\{\begin{array}{l} H_{a1}^{gA}-\varepsilon_{1,0}^{g}+\varepsilon_{a1}^{gA}\\ \vdots \\ H_{am}^{gA}-\varepsilon_{m0}^{g}+\varepsilon_{am}^{gA}\\ \vdots \\ H_{aM}^{gA}-\varepsilon_{M0}^{g}+\varepsilon_{aM}^{gA} \end{array}\right\}+\sum_{i}\beta_{ai}^{gA}X_{ai}^{gA}-\ln(t_{a}^{gA*}+1)=0,\;if\;t_{a}^{gA*}>0$$ (25)

$$\max\left\{\begin{array}{l} H_{a1}^{gA}-\varepsilon_{1,0}^{g}+\varepsilon_{a1}^{gA}\\ \vdots \\ H_{am}^{gA}-\varepsilon_{m0}^{g}+\varepsilon_{am}^{gA}\\ \vdots \\ H_{aM}^{gA}-\varepsilon_{M0}^{g}+\varepsilon_{aM}^{gA} \end{array}\right\}+\sum_{i}\beta_{ai}^{gA}X_{ai}^{gA}-\ln(t_{a}^{gA*}+1)<0,\;if\;t_{a}^{gA*}=0$$ (26)

where $H_{am}^{gA}=\sum_{i}\beta_{ami}^{gA}X_{ami}^{gA}+\ln\left(t_{m0}^{g*}+1\right)$. By assuming the error terms $\left(\varepsilon_{a1}^{gA},\cdots \varepsilon_{am}^{gA},\cdots ,\varepsilon_{aM}^{gA}\right)$ independently and identically follow a type-1 extreme value distribution, the *max* function is approximate to a *logsum* function.

$$\max\left\{\begin{array}{l} H_{a1}^{gA}-\varepsilon_{1,0}^{g}+\varepsilon_{a1}^{gA}\\ \vdots \\ H_{am}^{gA}-\varepsilon_{m0}^{g}+\varepsilon_{am}^{gA}\\ \vdots \\ H_{aM}^{gA}-\varepsilon_{M0}^{g}+\varepsilon_{aM}^{gA} \end{array}\right\}=\theta_{a}^{gA}\cdot \ln\sum_{m}\exp\left(\left(H_{am}^{gA}-\varepsilon_{m0}^{g}\right)/\theta_{a}^{gA}\right)+\varepsilon_{a}^{gA}$$ (27)

where $\theta_{a}^{gA}$ is the similarity parameter; $\varepsilon_{a}^{gA}$ is the error term of the allocated activity for the household-level time use. Then the new KKT first order conditions for the allocated activities can be obtained.

$$\varepsilon_{a}^{gA}=V_{a}^{gA},\;if\;t_{a}^{gA*}>0$$ (28)

$$\varepsilon_{a}^{gA}<V_{a}^{gA},\;if\;t_{a}^{gA*}=0$$ (29)

where $V_{a}^{gA}=\ln(t_{a}^{gA*}+1)-\sum_{i}\beta_{gi}^{gA}X_{gi}^{gA}-\theta_{a}^{gA}\cdot \ln\sum_{m}\exp\left(\left(H_{am}^{gA}-\varepsilon_{m0}^{g}\right)/\theta_{a}^{gA}\right)$.

HUF integrated with household task assignment involves two types of error terms, i.e. the error terms for imperfect substitutes and perfect substitutes respectively. Imperfect substitutes refer to activity participations and durations, namely, household time use, whereas, the perfect substitutes represent household task assignment, i.e. which household member is assigned to finish this ‘task’.

For household time use, in general, by assuming that all unobserved error terms in the household-level baseline utility functions for imperfect substitutes independently and identically follow a type-1 extreme value distribution, we can derive the probability of the optimal time use where the household member *m* undertakes the first $K_{m}^{S}$ of the *S* independent activities, and the first *K*^*J*^ of the *J* joint activities will be jointly undertaken by elderly couples, and the first $K_{m}^{a}$ of the *A* independent activities will be assigned to one of elderly couples. Above simple error assumptions are to derive the simplest version of multiple discrete and continuous choice model, which is MDCEV model. The MDCEV model can be extended to other more complex models by assuming more flexible error terms. For simplicity, the MDCEV model is employed in this paper to incorporate household task assignment and illustrate trade-offs among imperfect and perfect substitutes, as well as to allow for multiple constraints [29].

Given the error terms $\varepsilon_{0}^{g}=\left(\varepsilon_{1,0}^{g},\cdots ,\varepsilon_{m0}^{g},\cdots ,\varepsilon_{M0}^{g}\right)$, the probability derived from the household-level MDCEV-MNL model with multiple constraints [5] is:

$$\begin{array}{l} P(t_{1,1}^{gS*},\ldots ,t_{K_{1}^{S}1}^{gS*},0,\ldots ,t_{1M}^{gS*},\ldots ,t_{K_{M}^{S}M}^{gS*},0,\ldots ,t_{1}^{gJ*},\ldots ,t_{K^{J}}^{gJ*},0,\ldots ,t_{1}^{gA*},\ldots ,t_{K^{A}}^{gA*},0,\ldots |\varepsilon_{0}^{g})\\ =\left[\det(Q)|\varepsilon_{0}^{g}\times \prod_{m=1}^{M}\prod_{s=1}^{K_{m}^{S}}\frac{1}{\sigma }y(\frac{V_{sm}^{gS}|\varepsilon_{0}^{g}}{\sigma })\times \prod_{j=1}^{K^{J}}\frac{1}{\sigma }y(\frac{V_{j}^{gJ}|\varepsilon_{0}^{g}}{\sigma })\times \prod_{a=1}^{K^{A}}\frac{1}{\sigma }y(\frac{V_{a}^{gA}|\varepsilon_{0}^{g}}{\sigma })\right]\\ \times \left[\prod_{m=1}^{M}\prod_{s=K_{m}^{S}+1}^{S}Y(\frac{V_{sm}^{gS}|\varepsilon_{0}^{g}}{\sigma })\times \prod_{j=K^{J}+1}^{J}Y(\frac{V_{j}^{gJ}|\varepsilon_{0}^{g}}{\sigma })\times \prod_{a=K^{A}+1}^{A}Y(\frac{V_{a}^{gA}|\varepsilon_{0}^{g}}{\sigma })\right] \end{array}$$ (30)

where *y* is the probability density function of type-1 extreme value distribution for all activities; *Y* is the cumulative density function of type-1 extreme value distribution for all activities; *σ* is a scale parameter; $det(Q)|\varepsilon_{0}^{g}$ is the determinant of the Jacobian matrix **Q** conditional on the error terms of the “outside goods”. The Jacobian matrix **Q** can be obtained while using a change-of-variable technique to get the density of $\varepsilon_{1}^{g}=(\varepsilon_{1,1}^{gS},\cdots \varepsilon_{SM}^{gS},\varepsilon_{1}^{gJ},\cdots \varepsilon_{J}^{gJ},\varepsilon_{1}^{gA},\cdots \varepsilon_{A}^{gA})$ from the optimal time uses.

For the household task assignment, given the error terms $\varepsilon_{0}^{g}$ and the allocated activity *a* which will be undertaken (i.e. the duration of this activity which is from the household-level time use should be greater than 0), the conditional probability of assigned member *m*_*a*_ to undertake the allocated activity *a* can be formulated as a MNL model, since the error terms for household task assignment are assumed to follow an independent and identical type-1 extreme value distribution.

$$P(m_{a}^{}|t_{a}^{gA*}>0,\varepsilon_{0}^{g})=\frac{\exp\left(H_{am_{a}}^{gA}-\varepsilon_{m_{a}0}^{g}\right)}{\sum_{m}\exp\left(H_{am}^{gA}-\varepsilon_{m0}^{g}\right)}$$ (31)

By endogenously integrating the above two processes, a household-level MCDEV-MNL model with multiple constraints for household time uses and household task assignments conditional on the error terms $\varepsilon_{0}^{g}$ can be formulated. The probability derived from the household-level MDCEV-MNL model conditional on the error terms $\varepsilon_{0}^{g}$ can be formulated as Eq (32)

$$P(T^{g*}|\varepsilon_{0}^{g})=P(T^{g*}|\varepsilon_{0}^{g})\cdot \prod_{a}P(m_{a}^{}|t_{a}^{gA*}>0,\varepsilon_{0}^{g})$$ (32)

where, $T^{g*}=(t_{1,1}^{gS*},\ldots ,t_{K_{1}^{S}1}^{gS*},0,\ldots ,t_{1M}^{gS*},\ldots ,t_{K_{M}^{S}M}^{gS*},0,\ldots ,t_{1}^{gJ*},\ldots ,t_{K^{J}}^{gJ*},0,\ldots ,(0,..,t_{1m_{1}}^{gA*}..,0),\ldots ,(0,..,t_{K^{A}m_{K^{A}}}^{gA*}..,0),(0,\ldots ,0),\ldots )$.

The unconditional probability of the household-level MDCEV-MNL model can be formulated as follows.

$$P(T^{g*})=\int_{\varepsilon_{1,0}^{g}}\cdots \int_{\varepsilon_{m0}^{g}}\cdots \int_{\varepsilon_{M0}^{g}}P(T^{g*}|\varepsilon_{0}^{g})\prod_{m}f(\varepsilon_{m0}^{g})d\varepsilon_{1,0}^{g}\cdots d\varepsilon_{m0}^{g}\cdots d\varepsilon_{M0}^{g}$$ (33)

where $f(\varepsilon_{m0}^{g})$ is the probability density function of the error term $\varepsilon_{m0}^{g}$. A type-1 extreme value distribution can be assumed independently and identically followed by all error terms $\varepsilon_{0}^{g}$ for “outside goods”.

In terms of allocated activity, the probability of MNL-based household task assignment model can be formulated as follows.

$$P(m_{a}^{}|t_{a}^{gA*}>0)=\int_{\varepsilon_{1,0}^{g}}\cdots \int_{\varepsilon_{m0}^{g}}\cdots \int_{\varepsilon_{M0}^{g}}P(m_{a}^{}|t_{a}^{gA*}>0,\varepsilon_{0}^{g})\prod_{m}f(\varepsilon_{m0}^{g})d\varepsilon_{1,0}^{g}\cdots d\varepsilon_{m0}^{g}\cdots d\varepsilon_{M0}^{g}$$ (34)

The above-mentioned household-level time-use model is a revised household-level MCDEV model integrating household task assignment with multiple constraints, forming a household-level MCDEV-MNL model with multiple constraints. The household-level MCDEV-MNL model involving independent, joint and allocated activities not only tells “whether to participate in an activity” and “how much time will be spent on the activity”, but also provides discrete choices of “who will undertake the allocated activity”. For example, the model will provide a joint probability of assigning an allocated activity *a* to a household member *m*_*a*_, and giving $t_{am_{a}}^{gA*}$ time to the allocated activity.

In terms of estimation, a simulation-based maximum likelihood estimation method [5] is employed for the proposed household-level MCDEV-MNL model with multiple constraints. The log-likelihood function is formulated as follows.

$$L(\omega )=\sum_{g=1}^{G}\ln\left[\frac{1}{R}\sum_{r=1}^{R}P(T^{g*}|\varepsilon_{0}^{g})\right]$$ (35)

where the vector **ω** contains all unknown parameters required to be estimated. During the estimation process, the vector **T**^g*^ contains the observed time use of each household. The unknown parameters **ω** and error terms $\varepsilon_{0}^{g}$ will be drawn *R* times for each observation *g* to get the average probability.

## 5. Empirical analysis

### 5.1 Data

The data about household activity-travel information is extracted from the Travel Characteristics Survey (TCS) of Beijing, China, from Sep.1st to Nov.1st in 2014. Beijing is the capital of China, as well as the political, economic and cultural center of the whole country. Beijing had a permanent population of 21.54 million, and its economic development level was leading in the mainland, whose GDP had exceeded three trillion CNY in 2018 (National Bureau of Statistic, 2018). A typical residential registration system is implemented in Beijing, also known as *hukou*. The *hukou* is strongly attractive for people who would like to live in Beijing. With *hukou* in Beijing, an individual will acquire benefits from education, housing, social welfare, etc. By the end of 2016, there was up to 3.29 million elderly people (aged 60 and above) in Beijing, and the elder occupied about 24% of Beijing residents [59]. Many efforts have been made to provide a better welfare system and a more age-friendly living environment for the elder. Especially for the elder with *hukou*, more social welfares are enjoyed, such as the free bus program. More insights into the elderly population’s daily activity patterns in Beijing are expected.

The primary data sets record the daily activity-travel diaries information over a day 24h period (3am-3pm) of 101,815 family members from 40,003 households, which covers 0.52% population of Beijing (16 municipal districts). The information covers:(a) household attributes, including household structure and social economic attributes (income, own private car or not, residential district, etc.); (b) household members’ personal attributes, including age, gender, occupation, etc.; (c) household members’ trips, including travel origin and destination, travel mode, travel purpose, departure and arrival time, etc. Indirectly, activity duration can be obtained between the adjacent trips. In this paper, the elderly couples are the objects of study. Finally, the detailed data about activity participation and duration for 4743 elderly couples (aged 60 and elder) are utilized. The data is available in supporting information.

The descriptive statistics of elderly couples’ out-of-home activity participations and durations in workdays are shown in **Table 1**. In this paper, according to trip purposes and intra-household interactions among participants, the out-of-home activities are classified into work-related activity (independent), shopping (independent/joint/allocated), leisure (independent/joint), escort somebody (allocated/joint), personal business (independent), and others. Taking ‘shopping’ as an example, independent shopping means personal shopping, whereas allocated shopping means maintenance shopping to satisfy household-level demand. Joint shopping means the elderly couple goes shopping together.

**Table 1:** Descriptive statistics of household out-of-home activities.

	The elderly couples (observations)		Proportion (%)	Average duration (min)	Standard deviation (h)
*Household size*	4743				
The household with school children	363		7.7		
The household without school children	4380		92.3		
*Out-of-home Activities*	*patterns*	7820			
Work-related activity	independent	85	1.1	323.75	2.7
shopping	independent	212	2.7	56	0.82
	joint	1157	14.8	72.43	0.7
	allocated	846	10.8	67	0.73
leisure	joint	1191	15.2	112.29	1.12
	independent	2452	31.4	116.59	1.29
Escort	joint	151	1.9	34.7	0.37
	allocated	502	6.4	36.27	0.44
Personal business	independent	685	8.6	166.29	2.33
Others	independent	382	4.9	165.9	2.83
	joint	157	2.0	152.43	2.89

In total, there are 7820 observed activities conducted by elderly couples. Note that a joint activity for the couple is counted as 1. Among all observed activities, nearly 46% of observations are independent activities, where work-related activity has the smallest proportion (1.1%) and independent leisure accounts for the highest proportion (31.4%). It makes sense that the elderly population has more free time to enjoy recreations (e.g. strolling in the parks) due to retirements. In terms of joint activity, it occupies 32.1% of the observations. The elderly couples would like to go shopping or leisure jointly and enjoy the company of each other. Some evidences suggest that the longer time they spend together during non-compulsory activities, the more satisfactions they will gain [5,45].

Concerning the allocated activity, the proportion is about 17.2%, including allocated shopping and allocated escorting. The allocated shopping is undertaken by one of the elderly couple and aims at household maintenance. The allocated escorting is mostly related to escorting children for school. In China, impacts of school children on elderly couples are required to be paid attention to, since quite a number of Chinese elderly couples would like to help escort school children to release the young couples’ stress of juggling work and family. Among elderly couples who co-reside with school children, 57.3% elderly couples will take part in escorting activity, where 83.2% is escorting school children. For comparison, the escorting activity of elderly couples without school children is only 9.5%. Apparently, the school children have a great influence on the elderly couples’ daily time uses during workdays. An integrated analysis is necessary since the allocated activity comes from the interactions between household time use and task assignment.

Moreover, **Table 1** also describes the average durations of different activities. The largest time use is on work-related activity. The elderly workers experience less working time (about 5.4h) than younger commuters (about 8.56h) in Beijing. Except for escorting, the average duration of the other out-of-home activities are all larger than one hour.

Descriptive statistics of surveyed variables associated with elderly people are shown in **Table 2**. From the dataset, only 6.9% of the elderly people are employed; meanwhile, 34.2% of the elderly people are high-level educated (college above) and 95% of the elderly people have local *hukou*. In terms of the household with elderly people, the proportion of living with school children is 7.7% and the proportion of living in core districts [60] is 22.7%, while 17.3% of the households with elderly people have high income. Car and EB are owned by 25.4% and 22.2% households with elderly people respectively. More attention will be given in the following sections to analyze how these factors influence the elderly couples’ time uses and task assignments.

**Table 2:** Descriptive statistics of variables.

Variables	Observation	Proportion
***The elder people***	*9501*	
Sex (male = 1, female = 0)	4734	0.498
Age_ (>75:1)	1427	0.150
Local *hukou* (yes = 1, no = 0)	9031	0.950
Employment (yes = 1)	665	0.069
Education_(High:1)	3251	0.342
***Household with elderly people***	*4743*	
Income(>100,000CNY/year:1)	1647	0.173
Car ownership (1:> = 1)	2409	0.254
EB ownership (1:> = 1)	2114	0.222
Living with school children (yes = 1)	363	0.077
Core district (yes = 1, no = 0)	2158	0.227

Note: EB represents electric bicycle.

### 5.2 Estimations and discussions

The proposed household-level MDCEV-MNL model with multiple constraints is estimated based on the survey data. At last, specifications with the best goodness-of-fit are exhibited from **Tables 3**–**5**. All estimations can be accepted at least at a 90% confidence level (most estimations can be accepted at a 95% confidence level). The estimations which can be accepted at a 90% confidence level are marked by “*” in the tables. The scale parameter *σ* of the extreme value distributed errors in Eq (20) is estimated as 0.56 with a 95% confidence level. The satiation parameter for all activities is set as 1. For a simple illustration, the estimations, i.e. the specifications of baseline utility for independent, joint and allocated activities are separately displayed in **Tables 3–5**. The estimations on household task assignments are also displayed in **Table 5**.

**Table 3:** Specifications of baseline utility for independent activity.

	Attributes	Work-related	Shopping	Leisure	Personal business	Others
****Husband****	***individual***					
	Age(>75:1)	-1.502	-0.902	-0.903	-0.175*	-0.061
	Hukou(yes:1)	0.041*	0.026	0.002	0.051	0.040
	Education (High:1)	0.496*	0.007	0.003	0.023	0.023
	Employment	1.095	-0.223	-0.008	-0.005	--
	***household***					
	Income (>100,000CNY/year:1)	0.170	1.078	0.805*	0.049	0.047
	Car ownership(yes:1)	0.844	1.006	0.157	1.226	1.025
	EB ownership (yes:1)	0.025	0.865	0.207	1.509	5.091
	***Built environment***					
	Core district(yes:1)	1.115	0.797	0.780	2.052	0.855
	Constant	-6.574	-1.687	6.919	-2.925	-5.205
****Wife****	***individual***					
	Age(>75:1)	-0.963	-0.807	-0.802	-0.201	-0.233
	Hukou(yes:1)	0.032	0.004	0.189	0.006	0.009
	Education(High:1)	0.051	0.002	0.002	0.025	0.030
	***household***					
	Income(>100,000CNY/year:1)	0.237*	1.274	0.402	0.100	0.201
	Car ownership(yes:1)	0.448	0.223	0.068	0.050	0.057*
	EB ownership (yes:1)	0.367	0.596	1.135	1.108	0.204
	***Built environment***					
	Core district(yes:1)	1.259	1.001	1.356	2.007	0.431
	Constant	-1.755	-1.725	1.103	-1.020	0.302

**Table 4:** Specifications of baseline utility for joint activity.

Attributes		Shopping	Leisure	Escort	Others
***individual***					
****Husband****	Age(>75:1)	-0.204	-1.006	-1.036	-1.064
	Hukou (Yes:1)	0.026*	0.002	0.005	0.002
	Education(High:1)	0.003	0.007	0.025	0.030
****Wife****	Age(>75:1)	-0.207	-0.952	-1.004	-1.153
	Hukou (Yes:1)	0.303*	0.023	0.003	0.004
	Education(High:1)	0.004	0.002	0.037*	0.006
***household***					
Income(>100000CNY:1)		1.037	0.5021	0.052	0.011
Car Ownership(Yes:1)		1.024	1.5689	0.035	0.360
EB Ownership(Yes:1)		0.104*	0.6623	0.017	0.101
***Built environment***					
Core district(yes:1)		0.3027	0.5236	0.516	0.485
Constant		0.2013	1.262	1.479	1.570

**Table 5:** Specifications of baseline utility for allocated activity.

	Attributes	Shopping	Escort
***Household time use***	School Child (Yes:1)	--	0.703
	Core District (Yes:1)	3.679	2.026
	Constant	-1.978	-2.478
Scale parameter		0.569	0.670
***Household task assignment***	Sex (Wife:1)	0.154	0.441*
	Employment (Yes:1)	-0.240*	-0.502
	Car license (Yes:1)	0.305	1.003
	Can ride an EB(Yes:1)	1.629	1.450
	Constant(for husband)	-1.336	1.456

#### (1) Independent activity

The independent activity estimations show significances of work-related activity, independent shopping, independent leisure, personal business, and other independent activities, as shown in **Table 3**.

In terms of individual-related variables, age, *hukou*, education and employment are involved. The results show that the husband and wife aged 75 or above will have less out-of-home independent activities, perhaps due to more physical limitations. The *hukou* has positive impacts on leisure (only for the wife), shopping, personal business and other independent activities. With *hukou*, residents aged 60 or above enjoy ‘free bus’ in Beijing so that they would be encouraged to do more out-of-home activities. Additionally, education also has significant and positive impacts on work-related activity. It is perhaps because the elder with higher-level education is more likely to be rehired as short-time workers (e.g. the rehired professors or doctors). The employment has positive impacts on the work-related activity for the husband. In Beijing, the retirement age is usually 55 for the female and 60 for the male.

In terms of household-related variables, income, car ownership and electric bicycle (EB) ownership are significant. All of the three variables have positive impacts on out-of-home independent activities for both husband and wife, suggesting that the elder with higher household income, private car or EB are more likely to go outside. After all, out-of-home activities are monetary-and-energy consumed for the elderly population. The electric bicycle is a typical and special travel mode compared with developed countries and more attractive to the elder. Because electric bicycle costs less and is more labor-saving than the bicycle. It has become one of the most widely used travel modes in China with the inventory up to 250 million by 2019.

With regards to the built environment, the Beijing metropolis is divided into four districts [59] according to the density and accessibility. The core district attribute is significant and has positive impacts on out-of-home independent activities.

#### (2) Joint activity

**Table 4** shows that the significant specifications of joint activities include those of joint shopping, joint leisure, joint escort, and other out-of-home joint activities. The involved variables include individual-based attributes (age, *hukou* and education), household-based attributes (income, car ownership and EB ownership) and built environment (core district). Some special and interesting findings are discussed. The age has negative impacts on all out-of-home joint activities. It means although the couple can take care of each other, the older age will impede participation in joint activities. The education has positive impacts on joint leisure, escort and others, but the significances are relatively weaker. The car ownership has no effects on independent leisure for both husband and wife, whereas it is significant on joint leisure. The private car may be more convenient for the elder to go leisure together. For the leisure and other out-of-home joint activities, the independent participations can also be found, and for shopping and escort, the allocated activity type can also be found. Comparing the baseline utilities over the joint activities, the values of joint shopping and leisure are much greater than joint escort and others, indicating that the proportions of joint escort and others are much smaller than joint shopping and leisure, similar to the statistical results in **Table 1**. Perhaps this is also the reason that more attributes are not significant for joint escort and others.

#### (3) Allocated activity

The allocated activity is the integration of household time use and task assignment. The specifications of the two processes and the interaction between them are shown in **Table 5**.

From **Table 5**, significant specifications of allocated shopping and escort can be found. The estimations of the baseline utility for household time use represent whether the elderly couples will go shopping for household maintenance and escort somebody else respectively. And the estimations for household task assignment represent to whom of the elderly couple the household tasks of shopping and escort will be assigned. The scale parameters represent the endogenous interactions between household time use and household task assignment. In addition to constants, the variables like whether living with a school child, whether living in core district, sex, employment, whether having the car license, and whether can ride an EB are involved in the integrated model of household time use and task assignment for these two activities. Whether living with a school child has significantly positive impacts on escorting. As shown in statistics in “**5.1 Data**”, most of the escort activities are to escort school child. It means the elderly couples are more likely to take the household task of escorting school child when they live with school child in China.

In terms of household task assignment, the sex variable has positive impacts on shopping and escort, suggesting that the wife is more likely to undertake the household tasks. Some empirical evidences also show that female, regardless of their ages or employment status, takes on prime responsibilities for these tasks [19]. The employment shows negative impacts, indicating that the one employed hasn’t enough energy to consider both work and household tasks. The one with car license or who can ride an EB intend to do more household tasks since shopping or escort will become convenient if driving a car or riding an EB.

In a word, various factors will affect the elderly couple’s daily time use and task assignment. Due to the intra-household interactions, the same type of activity may have different activity patterns (independent, joint or allocated). For example, a part of the elder enjoys leisure independently, whereas others prefer spending leisure time with company. Additionally, most of the school children escorting are done by one of the elderly guardians from the same family, however there are still a few parts of the elderly couples would like doing escorting jointly (According to the statistical results from Table 1, the proportion of allocated escorting is 77.1%, joint escorting is 22.9%). In order to explore the impacts of the intra-household interactions, a direct comparison is inspired to see the extra gains or extra losses of task allocation comparing with independently or jointly participation, which will be discussed in “Net effects”.

### 5.3 The impacts of household task assignment on household time use

During the process of household task assignment, extra gains or extra losses may be generated between the elderly couples, since the one of the couple taking the household tasks means another one will have more time to attend other activities. Net effects are found in the literature to measure the extra gains or extra losses of the utilities of “whether participates in an activity”, namely the willingness to participations [5]. Since the allocated activity is derived from the integration of household time use and task assignment, this paper will employ the net effects to measure the extra gains or extra losses of the integrated utilities of “whether participates in an activity” and “to whom the household tasks will be assigned”.

The net effects can be formulated as the different baseline utility of allocated participation and joint participation.

$$Net_{a}^{gAJ}=\varphi_{a}^{gA}-\varphi_{a}^{gJ}$$ (36)

According to the third and fourth sections, the household-level baseline utility function of an allocated utility is shown as below.

$$\varphi_{a}^{gA}=\exp\left(\sum_{i}\beta_{gi}^{gA}X_{gi}^{gA}+\theta_{a}^{gA}\cdot \ln\sum_{m}\exp\left(\left(H_{am}^{gA}-\varepsilon_{m0}^{g}\right)/\theta_{a}^{gA}\right)+\varepsilon_{a}^{gA}\right)$$ (37)

**Fig 1:**
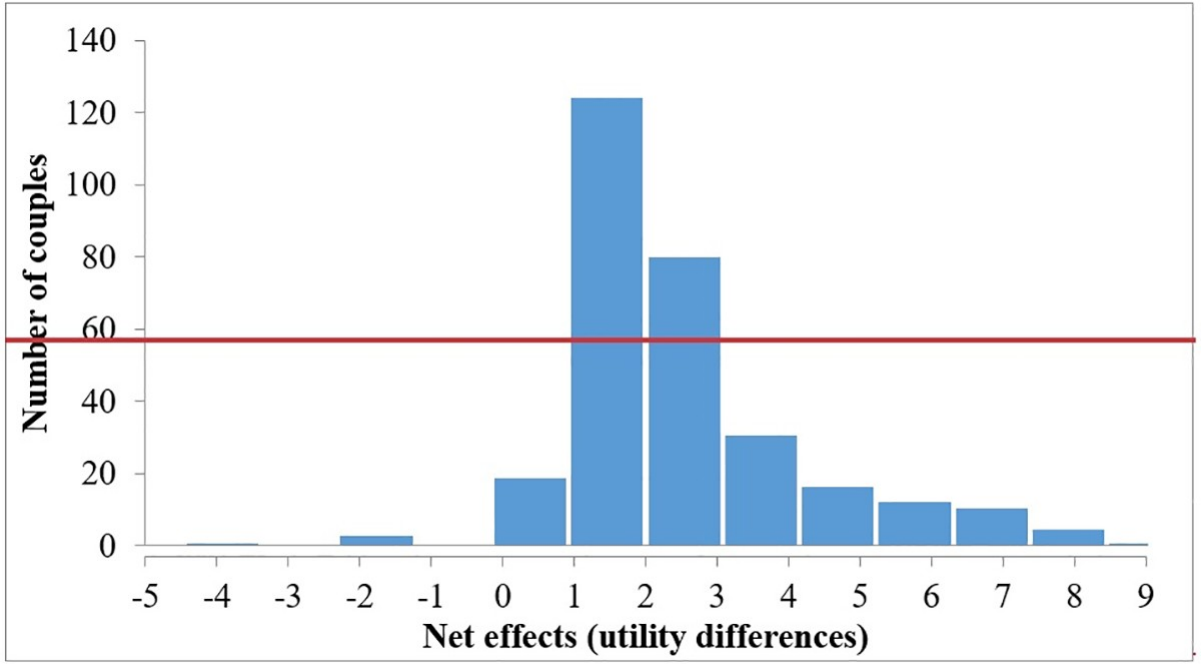
Net effects from the allocated escort compared with the joint escort.

The escorting activity includes escorting school children, which is a typical Chinese elderly couples’ contribution to the family. Most of the escorts are undertaken by assignment, whereas some of the escorts are undertaken jointly. Thus, the escorting activity will be taken as an example to illustrate the impacts of household task assignment on household time use by using above net effects of baseline utility. From the elderly couples with escorting activity, 300 couples are randomly selected to calculate the net effects. The distribution is shown in the Fig 1.

The mean value of the distributed net effects for 300 elderly couples is 1.58. The positive value means that the elderly couples are more willing to take the household task of escorting by assignment than jointly. By assignment, the one of the couple will be responsible for escorting so that another one will have more free time to do other activities. Then the total utility of the elderly couples will increase. The finding indicates that household task assignment will optimize household time use.

### 5.4 Policy implications

By the end of 2019, China’s elderly population aged 60 or above was up to 254 million, accounting for 18.1% of the total population. Population aging issues in China become more and more serious. Many efforts have been done to avoid elderly people’s social exclusions and achieve an active population aging society, including policies to encourage elderly people to undertake out-of-home activities. In Chinese context, elderly people would like to help busy young couples to take responsibilities for some household tasks which originally should be the tasks of young couples, such as shopping for maintenance or escorting school children, due to traditional family value affected by Confucianism. Especially in Beijing, high work pressures of young couples make them nearly have no time to do household tasks. Meanwhile, complex traffic environment and low level-of-service of school-bus in Beijing make escorting school children by adults necessary. In a word, many household tasks which should be the responsibilities of young couples are done by elderly couples. If elderly couples could be freed from those household tasks, they will have more time to do their own business. Household tasks which are energy-and-time-consumed might reduce the ‘quality’ of elderly couples’ out-of-home time-use patterns. In order to encourage the elderly population to participate in more out-of-home activities and reduce household tasks, some policy implications should be given based on model specifications.

1. Improve school-bus system. School-bus service could release the pressure of escorting, however, a hug gap still exists between the high demand and low supply of school-bus in China. Taking Beijing as an example, the huge city scale and potential safety factors weaken the attractions. Consequently, it’s suggested that more school-buses should be put in and the level-of-service of school-bus should be improved. Moreover, school buses should be given a higher right of way to improve safety factors and reliability.
2. Improve the social-cultural environment. The traditional family values drive the elderly people to undertake more household tasks, especially when three generations live in one roof. To encourage the elderly people to enjoy their own life and liberate from the household tasks, all of the household members should stand on his own feet and plan their daily activity routines more reasonable, for example, one of young couple should escort school children on the way to work.
3. Improve the EB environment. The results show that the EB ownership increases the participations and durations of elderly couples’ out-of-home activities. Given the safety factors, improvement of EB lanes is suggested to promote the EB ownership.

## 6. Conclusion

This paper proposed a framework of jointly modelling household time use and household task assignment with multiple constraints, where household time use is a multiple discrete-continuous choice problem while household task assignment is a discrete choice problem. The constraints are derived from each household member’s daily time budget. The integration of household time use and household task assignment are linked by allocated activities. On the one hand, an allocated activity competes for time resources with other activities given limited time budgets; on the other hand, decisions of an allocated activity participation are derived from household task assignment. In order to explore elderly couples’ time-use patterns affected by household task assignment, a joint household-level MDCEV-MNL model with multiple constraints is proposed and estimated by this paper.

In theory, the joint household-level MDCEV-MNL model with multiple constraints extends the joint individual MDCEV-MNL with a single constraint. The methodology and estimation algorithm for the proposed model enrich the area of joint discrete-continuous choice modelling.

In practice, the joint household-level MDCEV-MNL model with multiple constraints is customized in household time use and task assignment. An empirical analysis on elderly couples’ time-use patterns affected by household task assignment in Beijing of China represents the practical value of the proposed model.

Some major limitations and future expectations, but not the whole, are found in terms of the current research. For example, one limitation is that household time use is modelled by a simple structure of household-level MDCEV model. More complex model structures, such as group-decision based method, deserve to be formulated to further explore the interactions among household members. The second limitation is that household task assignment is simplified as a single discrete choice problem. In the next step, the study of household task assignment with more than one household member’s participation attracts the attempts. The third limitation is that travel-related information is less reflected. The household-level activity-travel patterns with household task assignment deserve deeper studies in the future.

## Supporting information

S1 Fig Net effects from the allocated escort compared with the joint escort. This figure shows the impacts of household task assignment on household time-use. Net effects measure the extra gains or extra losses of the utilities of “whether participates in an activity”, i.e. the willingness to participation.
(TIF)

S1 Table Descriptive statistics of household out-of-home activities. This table describes the statistical results of different activities.
(PDF)

S2 Table Descriptive statistics of variables. This table describes the statistical results of variables.
(PDF)

S3 Table Specifications of baseline utility for independent activity. (PDF)

S4 Table Specifications of baseline utility for joint activity. (PDF)

S5 Table Specifications of baseline utility for allocated activity. S3–S5 Tables shows the factors that affect the baseline utilities of various activities, where individual, household, built environment attributes are included.
(PDF)

S1 File Data. This is the data file we used in the research, which is uploaded separately.
(CSV)
