# Supplementary figures and images for: A joint model of household time use and task assignment for elderly couples with multiple constraints
Source: PLoS One. 2021 Mar 11;16(3):e0247187. doi: 10.1371/journal.pone.0247187 (PMC7951934; doi:10.1371/journal.pone.0247187)

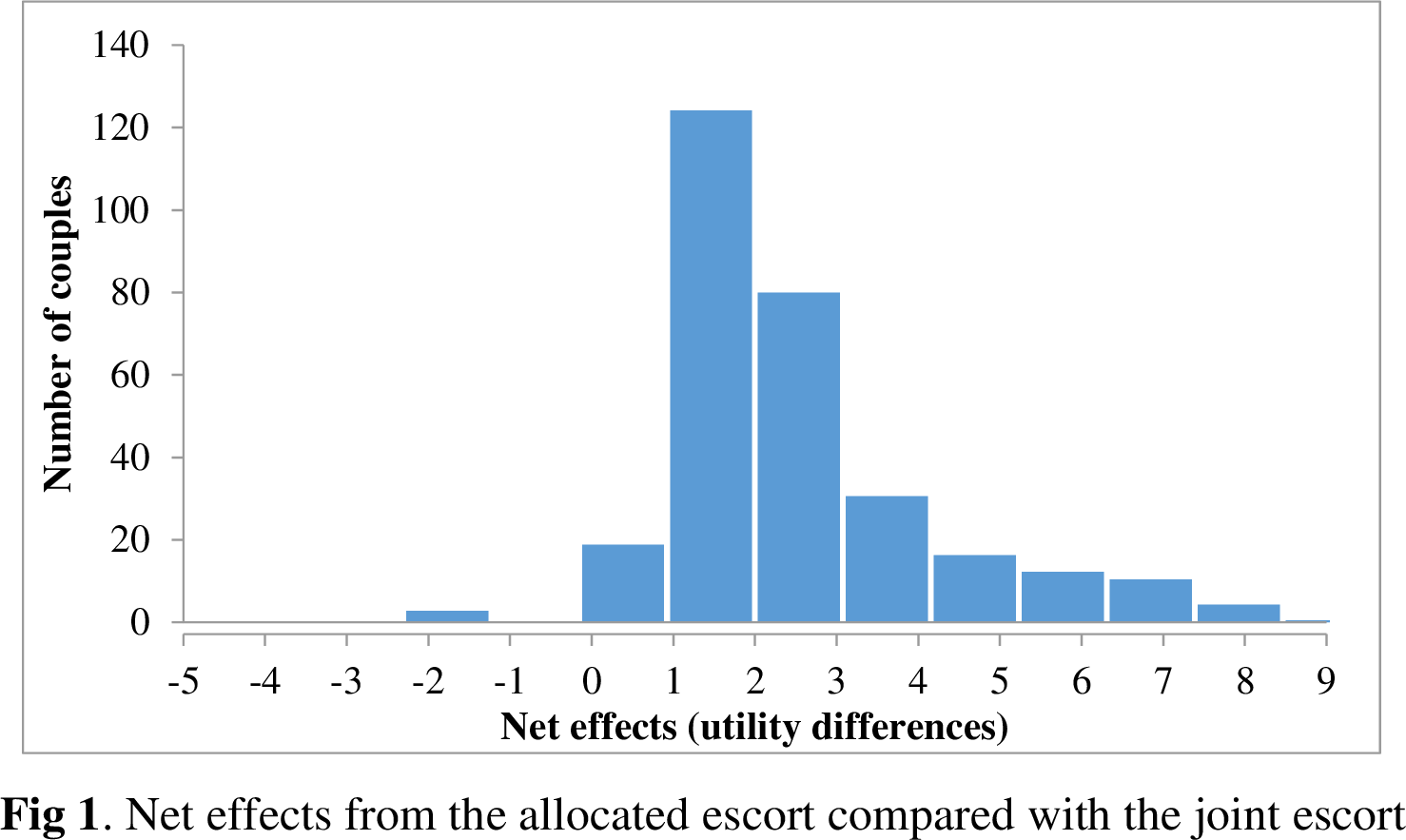

Supplement: S1 Fig — This figure shows the impacts of household task assignment on household time-use. Net effects measure the extra gains or extra losses of the utilities of “whether participates in an activity”, i.e. the willingness to participation. (TIF) [file pone.0247187.s001.tif]
