# Supplementary material for: A joint model of household time use and task assignment for elderly couples with multiple constraints
Source: PLoS One. 2021 Mar 11;16(3):e0247187. doi: 10.1371/journal.pone.0247187 (PMC7951934; doi:10.1371/journal.pone.0247187)
Supplement: S1 Table — This table describes the statistical results of different activities. (PDF) [file pone.0247187.s002.pdf]

**Table 1. Descriptive statistics of household out-of-home activities**

| | | The elderly couples (observations) | Proportion (%) | Average duration (min) | Standard deviation (h) |
|---|---|---|---|---|---|
| *Household size* | | 4743 | | | |
| The household with school children | | 363 | 7.7 | | |
| The household without school children | | 4380 | 92.3 | | |
| *Out-of-home Activities* | *patterns* | 7820 | | | |
| Work-related activity | independent | 85 | 1.1 | 323.75 | 2.7 |
| shopping | independent | 212 | 2.7 | 56 | 0.82 |
| | joint | 1157 | 14.8 | 72.43 | 0.7 |
| | allocated | 846 | 10.8 | 67 | 0.73 |
| leisure | joint | 1191 | 15.2 | 112.29 | 1.12 |
| | independent | 2452 | 31.4 | 116.59 | 1.29 |
| Escort | joint | 151 | 1.9 | 34.7 | 0.37 |
| | allocated | 502 | 6.4 | 36.27 | 0.44 |
| Personal business | independent | 685 | 8.6 | 166.29 | 2.33 |
| Others | independent | 382 | 4.9 | 165.9 | 2.83 |
| | joint | 157 | 2.0 | 152.43 | 2.89 |
