# Supplementary material for: A joint model of household time use and task assignment for elderly couples with multiple constraints
Source: PLoS One. 2021 Mar 11;16(3):e0247187. doi: 10.1371/journal.pone.0247187 (PMC7951934; doi:10.1371/journal.pone.0247187)
Supplement: S2 Table — This table describes the statistical results of variables. (PDF) [file pone.0247187.s003.pdf]

Table 2. Descriptive statistics of variables

Variables	Observation	Proportion
The elder people	*9501*	
Sex (male = 1, female=0)	4734	0.498
Age_ (>75:1)	1427	0.150
Local *hukou* (yes = 1, no = 0)	9031	0.950
Employment (yes=1)	665	0.069
Education_(High:1)	3251	0.342
Household with elderly people	*4743*	
Income(>100,000CNY/year:1)	1647	0.173
Car ownership (1:> = 1)	2409	0.254
EB ownership (1:> = 1)	2114	0.222
Living with school children (yes=1)	363	0.077
Core district (yes = 1, no = 0)	2158	0.227

Note: EB represents electric bicycle
