# Supplementary material for: A joint model of household time use and task assignment for elderly couples with multiple constraints
Source: PLoS One. 2021 Mar 11;16(3):e0247187. doi: 10.1371/journal.pone.0247187 (PMC7951934; doi:10.1371/journal.pone.0247187)
Supplement: S3 Table — (PDF) [file pone.0247187.s004.pdf]

S3 Table. Specifications of baseline utility for independent activity

	Attributes	Work-related	Shopping	Leisure	Personal business	Others
	individual					
	Age (>75:1)	-1.502	-0.902	-0.903	-0.175*	-0.061
	Hukou(yes:1)	0.041*	0.026	0.002	0.051	0.040
**Husband**	Education (High:1)	0.496*	0.007	0.003	0.023	0.023
	Employment	1.095	-0.223	-0.008	-0.005	--
	household					
	Income	0.170	1.078	0.805*	0.049	0.047

	(>100,000CNY/year:1)					
	Car ownership(yes:1)	0.844	1.006	0.157	1.226	1.025
	EB ownership (yes:1)	0.025	0.865	0.207	1.509	5.091
	Built environment					
	Core district(yes:1)	1.115	0.797	0.780	2.052	0.855
	Constant	-6.574	-1.687	6.919	-2.925	-5.205
	individual					
	Age (>75:1)	-0.963	-0.807	-0.802	-0.201	-0.233
	Hukou (yes:1)	0.032	0.004	0.189	0.006	0.009
	Education (High:1)	0.051	0.002	0.002	0.025	0.030
	household					
**Wife**	Income(>100,000CNY/year:1)	0.237*	1.274	0.402	0.100	0.201
	Car ownership(yes:1)	0.448	0.223	0.068	0.050	0.057*
	EB ownership (yes:1)	0.367	0.596	1.135	1.108	0.204
	Built environment					
	Core district(yes:1)	1.259	1.001	1.356	2.007	0.431
	Constant	-1.755	-1.725	1.103	-1.020	0.302
