# Supplementary material for: A joint model of household time use and task assignment for elderly couples with multiple constraints
Source: PLoS One. 2021 Mar 11;16(3):e0247187. doi: 10.1371/journal.pone.0247187 (PMC7951934; doi:10.1371/journal.pone.0247187)
Supplement: S4 Table — (PDF) [file pone.0247187.s005.pdf]

S4 Table Specifications of baseline utility for joint activity

	Attributes	Shopping	Leisure	Escort	Others
individual					
	Age (>75:1)	-0.204	-1.006	-1.036	-1.064
**Husband**	Hukou (Yes:1)	0.026*	0.002	0.005	0.002
	Education (High:1)	0.003	0.007	0.025	0.030
	Age (>75:1)	-0.207	-0.952	-1.004	-1.153
**Wife**	Hukou (Yes:1)	0.303*	0.023	0.003	0.004
	Education (High:1)	0.004	0.002	0.037*	0.006
household					
Income(>100000CNY:1)		1.037	0.5021	0.052	0.011
Car Ownership (Yes:1)		1.024	1.5689	0.035	0.360
EB Ownership (Yes:1)		0.104*	0.6623	0.017	0.101
Built environment					
Core district(yes:1)		0.3027	0.5236	0.516	0.485
Constant		0.2013	1.262	1.479	1.570
