# Supplementary material for: A joint model of household time use and task assignment for elderly couples with multiple constraints
Source: PLoS One. 2021 Mar 11;16(3):e0247187. doi: 10.1371/journal.pone.0247187 (PMC7951934; doi:10.1371/journal.pone.0247187)
Supplement: S5 Table — S3–S5 Tables shows the factors that affect the baseline utilities of various activities, where individual, household, built environment attributes are included. (PDF) [file pone.0247187.s006.pdf]

S5 Table Specifications of baseline utility for allocated activity

	Attributes	Shopping	Escort
	School Child (Yes:1)	--	0.703
Household time use	Core District (Yes:1)	3.679	2.026
	Constant	-1.978	-2.478

Scale parameter		0.569	0.670
	Sex (Wife:1)	0.154	0.441*
	Employment (Yes:1)	-0.240*	-0.502
Household task assignment	Car license (Yes:1)	0.305	1.003
	Can ride an EB(Yes:1)	1.629	1.450
	Constant (for husband)	-1.336	1.456
